# Proteomes Are of Proteoforms: Embracing the Complexity

**DOI:** 10.3390/proteomes9030038

**Published:** 2021-08-31

**Authors:** Katrina Carbonara, Martin Andonovski, Jens R. Coorssen

**Affiliations:** Faculties of Applied Health Sciences and Mathematics & Science, Departments of Health Sciences and Biological Sciences, Brock University, 1812 Sir Isaac Brock Way, St. Catharines, ON L2S 3A1, Canada; kcarbonara@brocku.ca (K.C.); mandonovski@brocku.ca (M.A.)

**Keywords:** proteomics, top-down, bottom-up, immunoassay, mass spectrometry, two-dimensional gel electrophoresis, Western blotting

## Abstract

Proteomes are complex—much more so than genomes or transcriptomes. Thus, simplifying their analysis does not simplify the issue. Proteomes are of proteoforms, not canonical proteins. While having a catalogue of amino acid sequences provides invaluable information, this is the Proteome-lite. To dissect biological mechanisms and identify critical biomarkers/drug targets, we must assess the myriad of proteoforms that arise at any point before, after, and between translation and transcription (e.g., isoforms, splice variants, and post-translational modifications [PTM]), as well as newly defined species. There are numerous analytical methods currently used to address proteome depth and here we critically evaluate these in terms of the current ‘state-of-the-field’. We thus discuss both pros and cons of available approaches and where improvements or refinements are needed to quantitatively characterize proteomes. To enable a next-generation approach, we suggest that advances lie in transdisciplinarity via integration of current proteomic methods to yield a unified discipline that capitalizes on the strongest qualities of each. Such a necessary (if not revolutionary) shift cannot be accomplished by a continued primary focus on proteo-genomics/-transcriptomics. We must embrace the complexity. Yes, these are the hard questions, and this will not be easy…but where is the fun in easy?


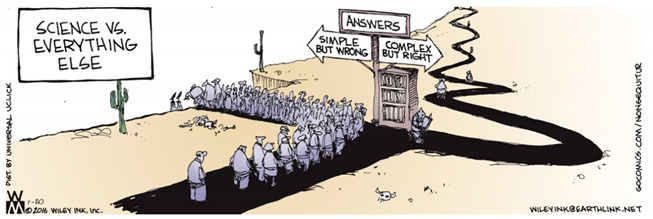


(**NON SEQUITUR © Wiley Ink, Inc.** Dist. By ANDREWS MCMEEL SYNDICATION. Reprinted with permission. All rights reserved.)

## 1. Introduction

Throughout a lifespan, the genome remains essentially unaltered in every somatic cell of an organism, the exception being epigenetic influences on gene expression, and hence proteins. In contrast, the proteome is constantly changing and responding to stimuli, both internal and external [1]. The proteome was first defined as, “the PROTEin complement expressed by a genOME” [2] but here we will emphasize how and why it is much more complex than this original definition implied. It is this complexity that must be respected and addressed to ensure the best possible analyses of proteomes.

**Figure 1:**
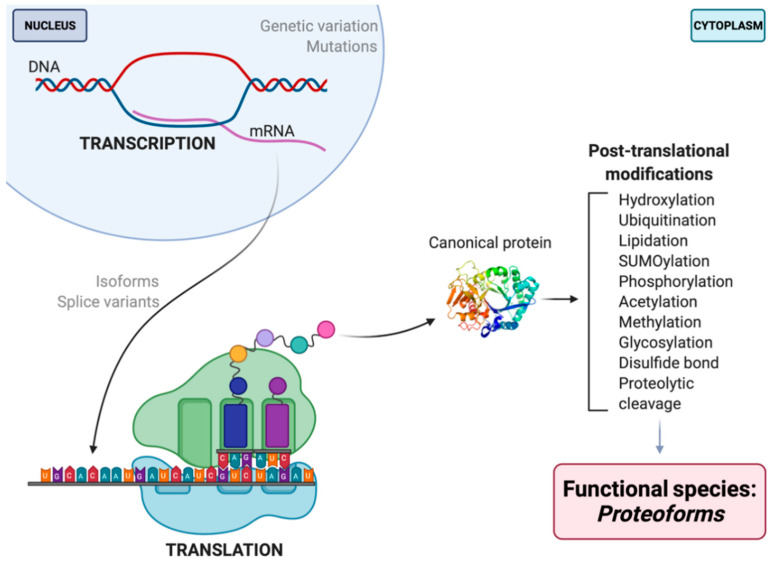
Schematic illustration of proteoform synthesis. Depicted are a handful (but not all) factors contributing to final proteoform configuration that are not seen in, nor predicted by, the central dogma. The PTM noted are but examples of the 100s of currently identified native modifications [23,24]. Each modification that occurs throughout the development and lifespan of a given amino acid backbone will yield multiple different proteoforms, each differing in their biological localization and/or function.

For the field of proteomics to effectively move forward, there must first be a discussion and broader consensus on what a ‘protein’ is. To what exactly does the word ‘protein’ refer? It is well known that proteins serve as antibodies, enzymes, messengers, and so much more [3]. However, the central dogma that we are so familiar with is as follows: DNA is transcribed into mRNA and then translated into an amino acid sequence, which is the primary structure of a protein [4,5,6]. Amino acids are thus the backbone of proteins, but they do not fully or effectively define its function, localization, or interactions. DNA/RNA coding sequence, mutations, and variability of translational start site are key elements that reveal information about primary protein structure and chemistry. These elements—along with the immense number of possible modifications that can occur at any given point before translation, after transcription, or in-between (e.g., RNA splicing, alternative splicing, alternate open reading frames (AltORF) [7,8,9], single nucleotide polymorphisms (SNP) [10,11], and mRNA editing [12,13,14,15,16,17]), post-translational modifications (PTM), and adducts)—each yield multiple different protein species of a given amino acid sequence, and each such species, or proteoform, differs in its biological function (Figure 1) [5,18,19]. Being the critical players in the majority of biological activities, the presence/absence or alterations in the abundance of given proteoforms can disrupt physiological functions, thus leading to disease phenotypes [20,21,22]. This makes proteoforms the most selective and useful indicators (e.g., biomarkers) of disease, remission, response to therapy, as well as the most specific targets for the rationale design of therapeutics.

This then raises the question of what exactly is a ‘protein’? In trying to address the complexity of the proteome, ‘protein’ is essentially a nonspecific term that generically refers to a canonical amino acid sequence (which is itself a proteoform). Proteoforms define function (or dysfunction) within an organism and drive cellular functions at the molecular level; therefore, one gene = one protein = one function is far from any longer being an acceptable logic [3,5,6,20,21,23,25,26,27,28,29]. The generic ‘protein’ term is thus useful for its breadth in referring to a class of molecules. As the genuine complexity of proteomes becomes increasingly more obvious, use of more specific terms—‘protein species’ or ‘proteoforms’—more accurately conveys the complexity being addressed, while ‘canonical protein’ more accurately describes only a common amino acid backbone.

While only the terms proteoform or protein species will be used here, there are other terms that have sought to deal with the limitations of the central dogma but only hint at the real complexity of proteomes. ‘Moonlighting proteins’ are defined as a class of multifunctional canonical proteins derived from a single gene that exhibit multiple biochemical and/or biophysical functions said to be due to changes in cellular localization, cell type, oligomeric state, PTM, or the cellular concentration of a ligand, substrate, cofactor, or product [5,30,31,32,33]. This does not include gene fusions, multiple RNA splice variants, or pleiotropic effects, although these can also modify the function(s) of a given canonical protein. Another term, ‘morpheeins,’ is used to describe proteins that change shape and assemble in alternate configurations with different functions [34,35]. Noncanonical open reading frames (ORF) have been the subject of intense investigation since their discovery, and their functional products are proving quite important [36]. An ‘alternative protein’ is defined as the product of an AltORF that does not match the sequence of the protein from a reference ORF of the same gene [7,8,9]. Additionally, ‘microproteins’ challenge the common definition of a gene, being functional entities arising from small ORF and consisting of ~10–100 amino acids [37,38,39,40]; these are missed in the standard definition of an ORF and thus of a canonical protein. Finally, the recent revelation of a reverse transcriptase activity in human cells (via Polymerase θ) may also indicate additional complications for genome and proteome analyses [41]. These are only a few of many observations that indicate the sheer complexity of proteomes and what actually constitutes a ‘protein’; it is crucial to incorporate all into our discussions and assessments if we are to genuinely address proteome complexity.

Currently, there are a number of methods that are used for proteomic analysis, and in an ideal world these would identify and quantify all proteoforms in a cell or tissue at a particular time and under a given set of environmental conditions [1,4]. However, as the complexity of the proteome is greater (and more dynamic) than that of the genome or transcriptome, deep analysis—routinely identifying and characterizing proteoforms—is much more difficult, particularly because amino acid sequences cannot be amplified like DNA/RNA.

Furthermore, proteomics does not assess protein expression, but rather the abundance of species or inferred canonical proteins depending on the methods used. Protein expression is difficult to quantify as there are conflicting data concerning the relationship between mRNA levels and that of the corresponding canonical proteins [42,43]. This is due to the fact that there are multiple processes beyond transcript concentration that contribute to establishing the amount of a given canonical protein present at any given time [44]. The presence of an mRNA tends to indicate the presence of the corresponding canonical protein however, it does not provide information as to what happens following transcription (e.g., proteolytic processing or other PTM, complexation) nor how quickly it may be degraded. Thus, whether a direct correlation exists between the levels of mRNA and the canonical amino acid sequences coded for, it seems unlikely—if not impossible—that any such correlation exists between mRNA and specific proteoforms. That is the critical point missed in all attempts to draw such correlations; conventional proteo-genomics/transcriptomics based on the central dogma does not address the actual biological complexity.

Simply, proteomes are complicated and multifaceted, and thus proteomic analyses must be as well: this must be recognized, appreciated, and respected. Simplifying the analysis and/or increasing its throughput does not simplify the issue. Thus, it must also be recognized and accepted that each analytical method currently applied in the study of proteomes has strengths and weaknesses, and that in some cases previously identified weaknesses have been effectively addressed while in others, weaknesses are only now being identified and must not be dogmatically perpetuated.

Our goal here is to critically evaluate the current state-of-the-art in proteomics: what are the pros and cons of available approaches, and thus what works and to what extent, what perhaps works less well or has pushed as far as it can regarding information, and where are improvements, refinements, or recalibrations needed if we are to fully develop a field that has the capacity to contribute as robustly as possible to systems biology [27,28,29,45]. We will also propose that the only way for proteomics to accomplish this is to be transdisciplinary. This includes understanding that proteomics is fundamentally based in analytical chemistry, operates at multiple disciplinary interfaces, and the need to integrate the numerous proteomic methods that currently exist to yield a unified discipline. There is also the need to better integrate proteomics with the other ‘omics’ to understand changes underlying disease and to identify rational biomarkers and treatment targets—but that is a discussion for another time [46,47].

While aspects of what we will discuss have been raised and reviewed over the years [1,3,27,28,29,48,49,50,51,52,53], here we seek to focus on the integrated assessment of proteomics as a whole—indeed, its need for integration—beginning with questions concerning terminology used. Our goal is to broadly consider the sheer complexity of proteomics and how this is addressed by current operating philosophies and associated analytical approaches. Yes, these are the ‘hard’ questions, but they must be addressed as directly, transparently, and realistically as possible to ensure that the field can move most effectively forward with routine quantitative assays that address the depth and breadth of proteomes as well as the need for effectively targeted analyses.

## 2. What Is a Proteome?

The definition of a proteome differs depending on who you ask. For some, the sum of all protein species during the entire lifespan of an individual is considered the ‘proteome’ [4], while others believe it is the sum of proteoforms expressed in a select biological material at a given time (e.g., specific tissue, cell type, organelle, or fluid) [50]. While both are reasonable, a consensus on a definition would be useful as concise and consistent language is key to the clearest, most unambiguous communication. The literature is replete with reports of the ‘total’ proteome analysis of a particular sample (e.g., blood, brain, and saliva) when only a sub-fraction of the proteome was investigated (i.e., specific ranges of molecular weight (MW)/isoelectric point (p*I*) [i.e., 3–20 kilodalton (kDa), p*I* 6–10], soluble fraction, and/or only canonical proteins). There must be clarity in defining what is being analyzed. If we are to adopt the definition, “*all protein species during a lifetime,*” [4], then there is currently no lab nor methodology that has or can effectively identify and quantify the human proteome, let alone that of any other species. Perhaps, then, the definition, “*the sum of proteoforms expressed in a selected type of biological material at a certain time,*” [50], is more appropriate from a purely practical standpoint, if perhaps not yet fully technologically feasible. Sub-proteome investigations would thus constitute any analyses focusing on only a select portion of the total sample (e.g., total membrane, total soluble, or <40 kDa).

Since completion of initial human genome sequencing in 2001, a number of studies have demonstrated numerous errors and confirmed that a large number of sequences (~8–10%) are missing from the reference genome (to some extent possibly due to inherent bias) [54,55,56,57,58,59]. This also does not take into consideration the ongoing identification of new AltORFs and small ORFs. As there is still no firm consensus on the size of the human genome, and the number of protein-coding genes is currently thought to be ~19,000–20,000, it is difficult, at best, to reasonably estimate the size of the human proteome [23,56,59,60,61]. Notably, this number also does not include regions coding for alternate RNA species (e.g., long non-coding and micro-RNA) that in many cases also affect canonical protein levels. If the one gene = one protein hypothesis were correct, then there should be a fixed number of identifiable, unmodified (canonical) human proteins. However, this simple definition is inconsistent with the presence of variants of those canonical proteins.

It is therefore not surprising that attempts to estimate the number of proteoforms within the human proteome range anywhere from 98,000 to 6 million, and even >1 billion potential species [5,23,50,61]. Nonetheless, much research continues to only infer the presence of canonical proteins. Despite the uncertainty of how many proteoforms exist within the human proteome, there are methods to determine the variety and quantity of proteoforms present in different samples, although not yet with the sensitivity to fully address the potential dynamic range of the species present (i.e., one copy—a few copies—millions of copies). Rather than despairing over this or bemoaning the fact that granting agencies simply cannot afford—for both financial and logical reasons—to put the latest new mass spectrometer into every lab, every year, we must capitalize on the techniques, instrumentation, and analyses that are proven, and open to even further improvement. We must embrace the complexity of proteomes by continuously striving for better, deeper analyses.

## 3. Proteomics

Proteomics is thus defined as the systematic analysis of proteoforms that constitute a given proteome, including their diverse properties. However, this is by far not a consensus definition considering that the bulk of current published research seeks to assesses or infer the presence of only canonical amino acid sequences as opposed to proteoforms. Nevertheless, with the working definition presented, the aims of proteomics as a discipline are to provide accurate, reliable, and detailed descriptions of the proteoforms present and their functions, thereby providing rational insight into the molecular mechanisms underlying biological systems in both health and disease [3,4,27,28,29,51,62,63].

At a practical, day-to-day research level, the main goal of proteomics is to identify and quantify all the proteoforms present in, for example, a cell or tissue—a goal that has yet to be achieved for perhaps any species. For this to be accomplished, sequence, quantity, nature and state of modification, interactions with other proteoforms/molecules, activity, subcellular distribution, and structure of every proteoform would have to be known [1,5,62]. While this is not currently possible, many different approaches have been designed to assess proteomes, at least at the compositional level, although some have only a limited focus on proteoforms, or on only a single type of PTM (e.g., phosphorylation).

**Figure 2:**
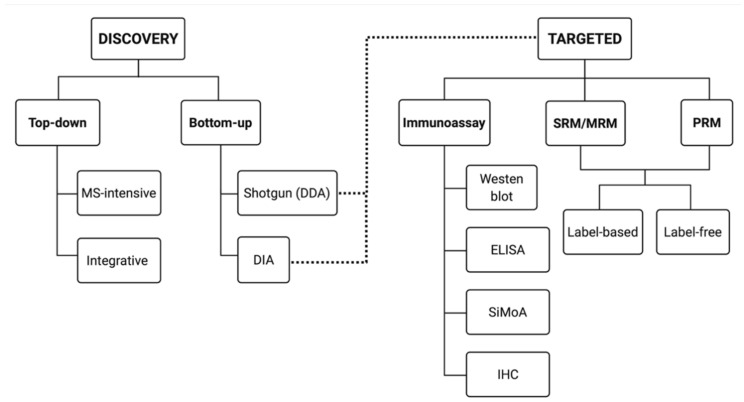
Proteomics: discovery and targeted approaches. Discovery proteomics is defined by two main approaches: top-down (resolution of intact protein species) and bottom-up (peptide mass spectrometry (MS) of proteolytic digests). Targeted proteomics involves either antibody- or MS-dependent approaches. Data dependent acquisition (DDA) and data independent acquisition (DIA) were initially developed for discovery but can be modified to also serve in a targeted approach.

Currently, there are two general approaches to address proteome composition: ‘discovery’ or a ‘targeted’ (Figure 2). Discovery proteomics is exploratory, untargeted—hopefully unbiased—and aims at mapping a given proteome or sub-proteome as deeply as possible without any preconceived notions as to what exactly will be found beyond differences between two or more conditions. Using the identifications made in a discovery approach, a targeted approach is critical for validating identified protein species of interest on a larger scale (i.e., validation of potential biomarkers). Thus, currently, targeted proteomic workflows generally involve the selective detection and assessment of particular canonical proteins of interest, hopefully with high sensitivity, quantitative accuracy, and reproducibility [64,65,66,67].

However, if the discovery approach has misidentified a particular proteoform or change in abundance, then the targeted approach will produce contrasting results, or vice versa. For example, a study identifies phosphorylated protein X to be significantly linked in causation of a disease. An antibody-based approach (Section 5.2) is then used to validate this result but finds that there is no statistically significant link between total detected canonical protein X and the disease condition. This occurs because a discovery proteomic method was used to identify a specific proteoform but the ‘standard’ validation attempt in a targeted approach usually only identifies total canonical amino acid sequences. This highlights the need for discovery ***and*** targeted proteomics to be as sensitive, accurate, and integrated as possible. Unless both approaches work in tandem, with the same definitions and objectives, they can simply confound one another.

## 4. Discovery Proteomics

**Figure 3:**
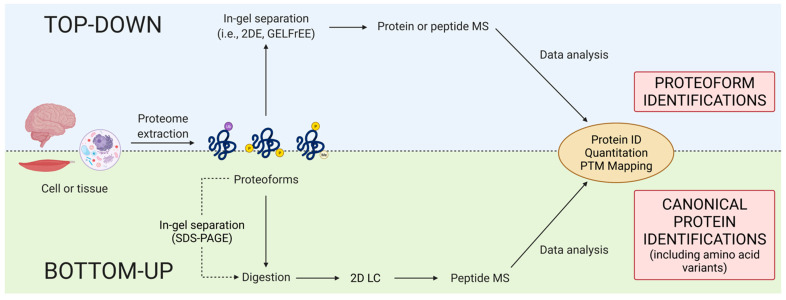
Top-down versus bottom-up proteomics. This schematic depicts a general description of the workflows for these two discovery approaches. While both rely on final MS analysis for identifications (not to oversimplify the analysis of intact proteoforms), the main differences lie in the up-front analytical approaches. Top-down resolves intact proteoforms prior to MS while bottom-up generally bypasses any initial separation technique. Thus, top-down provides proteoform information while bottom-up can only provide (limited) amino acid sequence information. Nonetheless, perhaps the most important point to immediately emphasize is the critical importance of high quality/high resolution MS to proteomics as an integrative discipline, now and into the future.

Within discovery proteomics, there are two general analytical approaches: top-down and bottom-up (Figure 3). There is, however, a notable discrepancy concerning the definition of top-down. Some use purely instrumentation-centric definitions based solely on what enters the mass spectrometer, ignoring the importance and indeed use of upfront separations/prefractionations in determining that [68]. Noting again that proteomics is analytical chemistry [28], we emphasize that, following proteome extraction, top-down approaches resolve intact proteoforms prior to their identification (e.g., by mass spectrometry [MS] or immunoblotting) whereas bottom-up methods use peptides generated from a gross proteolytic digestion of the extracted proteome as surrogates of the original intact proteoforms to only *infer* the presence of *potentially intact* canonical proteins [3,69]. The bottom-up method thus provides a rapid scan to identify likely canonical proteins but lacks the capability of routinely providing critical information concerning the myriad of proteoforms that define proteomes. 

Nonetheless, this bottom-up approach has enabled the Human Proteome Project (HPP) of the Human Proteome Organization (HUPO) to recently announce an almost complete (~90%) cataloging of canonical proteins corresponding to known/accepted gene coding regions [70,71]. Conservatively, an estimated 1899 canonical proteins remain to be identified; what that corresponds to in terms of proteoforms is anyone’s guess. This critical update thus qualifies that biologically functional proteoforms have not been yet identified due to the difficulty in their measurement, and future projects will thus also be needed to incorporate heterogenous RNA splicing, PTM, and single amino acid variants [70]; we suggest that international efforts should also include alternate proteins [7,8,9] and very small coding regions [8,72]. Additionally, researchers will need to be cognizant of ongoing developments in genome analysis and incorporate them into any future analyses. It is also now clear that some previously identified genes do not code for amino acid sequences, and thus these do not in fact yield proteins. Thus, while this new catalogue of canonical protein sequences is indeed a critical milestone, it is the minimally essential step forward in terms of a fundamental tool to now undertake the necessary critical deep analytical dive(s) into proteomes.

### 4.1. Bottom-up

**Figure 4:**
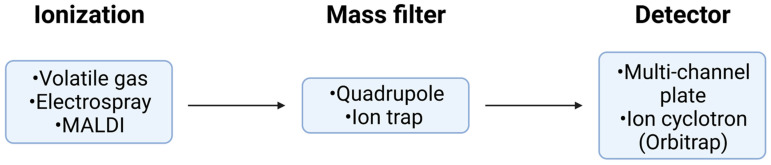
Schematic of MS/MS. A basic overview of the four main systems of MS/MS and the different methods for each. Peptides undergo separation via LC prior to ionization. Peptides are then transformed into ions before entering the mass filter where precursor ions are then selected prior to collision-induced dissociation. The resulting fragment ions are then separated and transmitted to the detector. The mass filter measures the mass of the ions and the detector counts the ions. This information can then be combined to determine the mass-to-charge ratio (*m*/*z)*, leading to identification of a peptide.

In recent years, bottom-up has come to dominate the field of proteomics [27,52,73,74]. The main reasons for the preferred use of bottom-up over top-down are *claims* of high throughput, ability to produce large volumes of raw data, and improved sensitivity, as well as the refrain that it is ‘faster’ and ‘easier,’ although, even if true, those are perhaps not ideal reasons to select an analytical approach. This approach—colloquially known as shotgun proteomics—combines liquid chromatography (LC) and tandem MS (MS/MS) to analyze peptide mixtures obtained from total proteolytic digests of proteome extracts (Figure 4). Data dependent acquisition (DDA) methods are predominantly used to identify canonical proteins, automatically selecting precursor ions from those detected in a survey scan. The approach is designed to select precursor ions in order of decreasing relative abundance while requiring a minimum threshold of abundance [21,25,66,75]. The identified peptides are then matched to a corresponding canonical protein (i.e., amino acid sequence) by searching protein sequence databases [76]. While peptide MS is an extremely powerful technology, there are several concerns with its application. 

The most noted is that hydrophobic peptides tend to stick to LC columns and also produce poor MS signals when routine soft ionization techniques (e.g., electrospray ionization [ESI]) are used. Thus, improving the coverage of hydrophobic proteins has been one critical area of focus. DDA methods also tend to be biased toward the most abundant proteins, making them vulnerable to high sample complexity and/or dynamic range [21,25]. Overall, the bottom-up approach is purely assumptive as it identifies proteolytic peptides and from those, infers the primary structure of a presumably intact canonical protein; however, it therefore does not actually sequence proteoforms [6,63,77]. Thus, the real question for bottom-up analyses is ‘how many corresponding proteoforms does the apparent change in total abundance of a putative intact canonical protein actually represent?’ This is further complicated as there is no general consensus on the number of peptides needed for a positive identification of a canonical protein [6,28,76,78,79]. Criteria seemingly vary from lab-to-lab (and journal-to-journal). Thus, substantial sequence coverage—some argue, with solid rationale, that only 100% coverage is optimal [5] or at least several peptides that roughly cover the full span of the sequence (i.e., minimally near the C and D termini and the middle)—is required to confidently assume a canonical protein identification [28,80]. We suggest using a minimum of three peptides, that span the range of the apparent canonical protein sequence, as criteria for a positive identification: this is further strengthened if proteoforms have first been resolved (i.e., top-down analysis) [27,28,81]. Indeed, there are many claims in the literature of canonical protein identifications that are based on very limited data (i.e., one or two peptides), making the inherent assumptions of even greater concern [27,82]. Notably, manufacturers (who heavily influence directions in proteomics research) promote new MS instruments based on the weakest possible sequence criteria and thus, each year claim a certain increase in the number of peptides and thus supposedly canonical protein identifications. However, these claims omit proteoform identifications. 

**Figure 5:**
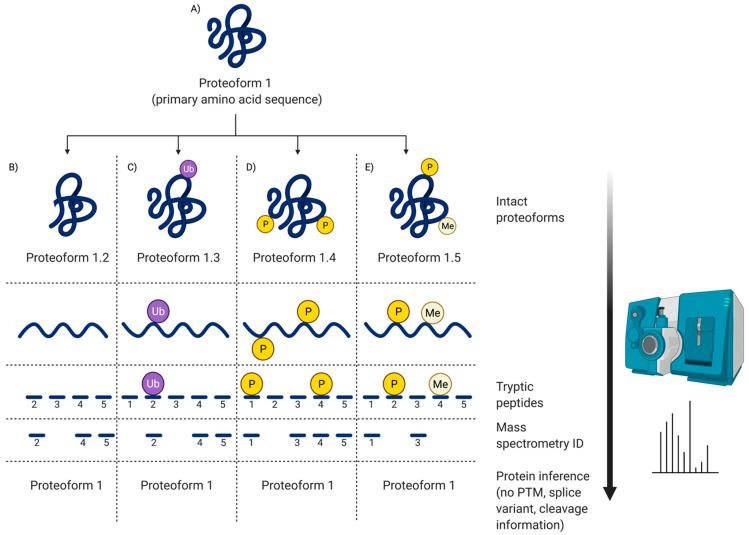
Peptide MS. This illustrates the information obtained via routine peptide MS. (**A**) Canonical protein (primary amino acid sequence); (**B**) PTM = Proteolytic cleavage; (**C**) PTM = Ubiquitination; (**D**) PTM = Two phosphorylations; (**E**) PTM = Phosphorylation and methylation. As only peptides are being sequenced, the ‘canonical protein’ identifications are based on inference; thus, as shown in (**B**), even though there has been a native proteolytic cleavage to generate another proteoform (i.e., likely to modify the biological activity of the canonical protein—Proteoform 1), it will not be detected by inference identification. Notably, other than potentially identifying SNP, no proteoform information is obtained via peptide MS without specific additional processing and assays.

Thus, one of the main complications that arises is a lack of standardized methods and thus poor reproducibility between different laboratories [80,83]. Regrettably, this has likely been a problem since the first SDS-PAGE gel was resolved in the *second* lab to ever try the method. In the case of identifying canonical proteins from available databases, a key example of this issue is an independent and unbiased study involving 20 highly purified recombinant proteins (expressed in *E. coli*), and that contained at least one unique tryptic peptide of 1250 ± 5 Dalton (Da) [76]. This sample was sent to 27 independent MS laboratories with the task of identifying all 20 canonical proteins and unique peptides using their routine procedures and instrumentation. Only seven labs identified all 20 proteins and only one lab reported all tryptic peptides of 1250 Da. Of the other 20 labs, the list of identified peptides differed and there was a general inability to identify even highly purified proteins in such a low complexity sample; the issues were largely attributed to differing protocols and methods across labs, as well as stochastic and irreproducible ion selection [76,84]. When not carried out using stringent protocols and criteria, in particular routine technical replicates, this approach has shown a lack of reproducibility, yielding inconsistent results even when analyzing the same sample, as well as high rates of false positives and false negatives [5,76,79,85,86]. Additionally, it is difficult to identify variants/modifications with routine peptide MS as these peptides are most often of lower abundance and more difficult to identify from their fragmentation spectra compared to nonmodified peptides (Figure 5) [82,87]. It is important to note that while it is entirely feasible to identify PTM on peptides, it is far from a quick and easy task (Section 4.3), and certainly confounds the promotion of shotgun analyses as ‘high-throughput’ (not to mention requiring appropriately sensitive instrumentation) [27].

Furthermore, any attempt to quantify proteoforms in such bottom-up analyses would be impossible considering that all information essential to doing so is lost upon the initial gross proteolytic digestion of the sample. Thus, as widely noted, the absence of a canonical protein from the list of those identified does not indicate the absence of the protein from the sample (nor, more importantly, select, low abundance, proteoforms) [66]. In all proteomic analyses, the concern is always what is missed—might it be the key player in the mechanism or condition being studied? This would thus also appear to be the current issue with newer approaches to protein/peptide sequencing (e.g., nanopores and DNA-PAINT), which focus on amino acid sequences but not PTM [6,88].

Seeking deeper analyses, data independent acquisition (DIA) methods have been used as an alternative to DDA. These methods either acquire fragmentation spectra of the entire mass range simultaneously or set predefined windows to cover the whole mass-to-charge ratio (*m*/*z*) ranges of proteolytic peptides, thereby eliminating the ‘one peak at a time’ selection process used in DDA [21,25,89]. All the peptide mass ranges within this window are acquired without pre-selection, leading to an unbiased fragmentation spectrum record of the complete set of peptide precursors of a given sample [21,84]. A popular variant of this method is Sequential Window Acquisition of all Theoretical Mass Spectra (SWATH-MS). SWATH-MS fragments ionized peptides systematically using large precursor isolation windows and records all fragment ions simultaneously, yielding high specificity identification of canonical proteins [84,90,91]. This method has numerous advantages including decreased fragmentation spectra complexity, improved precursor ion selectivity, and increased proteome coverage. While it is low cost and requires simple sample preparation (in comparison to label-based methods, Section 5.3.1), there is a strong need for standardization of equipment and protocols between laboratories [21]—an issue so true of all techniques used to analyze proteomes. Thus, while it is possible to use SWATH-MS to create fragment ion maps of all MS-measurable peptides in a proteome that can be used universally to analyze and compare samples in silico [90], lack of standardized operating procedures severely limits the likelihood of consistent and comparable data between different lab groups. Additionally, there are some claims that SWATH-MS is slightly limited in terms of sensitivity and dynamic range, indicating the need for further refinements before it can be used most effectively for biomarker discovery and validation [21,84].

Thus, while bottom-up may serve as a rapid, low-resolution scan of a proteome, it enables deep, high-resolution proteome analyses when paired with two-dimensional gel electrophoresis (2DE). The 2DE protocol was developed in 1975, essentially initiating the discipline of proteomics [92,93]. Since inception, this method has also received its share of criticism, mainly around original issues concerning the resolution of low abundance proteoforms with extreme p*I* and MW, and hydrophobic protein species [73,94,95]. Most notable, however, being a mature technology, these issues have been addressed and the approach has been substantially refined over the last two or more decades to address purported shortcomings (Section 4.2.2). Unfortunately, many of the negative claims that still appear in review articles have simply become dogma, often perpetuated by those who have no experience with the technique, nor certainly reviewed the relevant primary literature from at least the last twenty years [1,29,49,81,96,97,98,99,100,101,102,103,104,105]. Simply, refined 2DE and its modifications can effectively resolve many (hundreds of) thousands of proteoforms across a broad range of classes and physico-chemical characteristics (i.e., soluble, membrane, acidic, basic, large, and small) including those of low abundance, and do so in parallel technical replicates [27,29,81]. Like bottom-up approaches, the biggest issue with the method is likely the lack of consistent protocols between labs, making unified gel databases somewhat untenable unless direct and routine calibration of both p*I* and MW are implemented.

### 4.2. Top-Down

#### 4.2.1. Integrative

**Figure 6:**
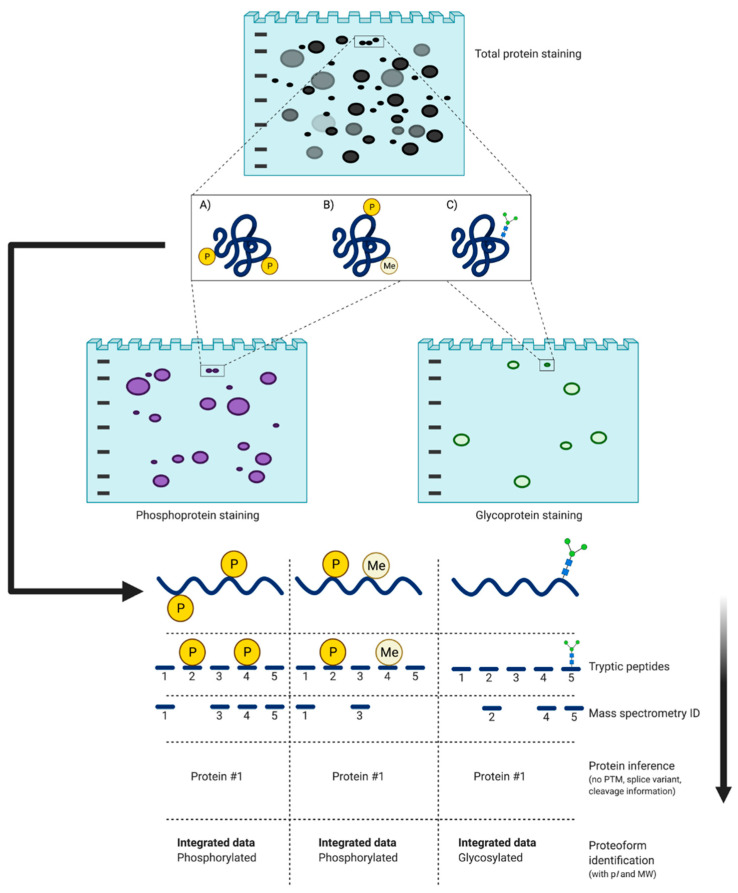
Integrative top-down proteomics via 2DE and PTM post-staining. (**A**) PTM = two phosphorylation sites; (**B**) Phosphorylation and methylation; (**C**) Glycosylation. Different PTM can change the p*I* and MW of a protein species thus, altering its final resolution in a 2D gel, which can be seen using a total staining method. Additional selective staining (e.g., phospho- and glyco-protein staining) can be used to identify these proteoforms prior to digestion and MS. Phosphorylation yields more acidic species and sugar groups increase MW [113]. Typically, a chain of protein species as seen in the 2D gel is often indicative of an identical canonical protein with varying modifications.

There is currently only one high resolution and high sensitivity method that can provide a genuinely deep assessment of proteomes at the critical level of proteoforms: 2DE coupled with LC/MS/MS [1,27,28,29,52]. The first dimension of 2DE is isoelectric focusing (IEF) which separates protein species according to their p*I*—the specific pH at which the net charge of the proteoform is zero [106,107,108]. Following IEF, species are then resolved by size (i.e., nominally and MW) via sodium dodecyl sulfate polyacrylamide gel electrophoresis (SDS-PAGE) [92,109]. The importance of the two separate dimensions lies in the fact that canonical proteins speciate into proteoforms that differ in MW and/or p*I*, and thus resolve into different spots on the gel, despite having identical (i.e., canonical) amino acid backbones (Figure 6). Following staining and quantitative image analysis, select protein spots are excised from the gel and proteolytically digested prior to LC/MS/MS [81,98,102,104,105,110,111,112]. Selective staining, deep imaging, and third-dimension separations (3DE) may also be employed to further improve resolution (Section 4.2.2). 

Many choose to bypass this method, supposedly due to the fact that it requires manual dexterity and demands technical precision. Strangely, this hackneyed comment not only describes most if not all scientific techniques but, again, tends to generally appear in reviews written by those with seemingly little to no 2DE experience. While bottom-up is praised for ostensibly being a high-throughput technology, it requires its own fair share of time, manual dexterity, and technical precision in packing, optimizing, and subsequently effectively flushing/cleaning different LC columns, adjusting fittings, fixing pumps, and cleaning clogged electrospray systems, not to mention the need to run *sequential* technical replicates (assuming this is even being done, since parallel replicates are not possible). Simply trying to improve LC resolution has been a major topic for decades, including the need for better columns [114,115]. It would thus behoove researchers using MS core facilities or commercial services to fully understand exactly how the analysis of their samples was conducted and whether the shotgun data they receive are based on technical replicates. This begs the question: does this approach truly require less time and technical input or are many researchers simply unaware of the issues associated with it? Overall, then, we respectfully suggest that the focus should be on the quality of the data that can be delivered, and a willingness to invest the time to acquire the best possible data at each stage of analysis. *It is not the rate or volume of data generated but rather the quality that ultimately matters*.

#### 4.2.2. 2DE: Addressing the Dogma

Extraction of membrane (i.e., hydrophobic) proteoforms has long been considered an issue with 2DE sample buffer. This is in part heavily dependent on effective sample handling before extraction [97]. Thus, while it has been established that hydrophobic proteins are effectively resolved by 2DE, their detection in-gel is often ‘swamped’ by the more abundant soluble proteoforms. It would thus seem that contrary to two decades or more of dogma, the real issue with 2DE is proteoform detection rather than resolution [81,99,100,102,103,116,117]. Again, the maturity of the method has yielded effective solutions. 

There are numerous stains that may be used for total proteome detection. While popular, SYPRO Ruby, a sensitive, easy to use, and fully MS compatible fluorescent stain, is quite expensive (as are variants that have appeared since its release ~20 years ago) [99,116]. To address this, a colloidal Coomassie Brilliant Blue (cCBB) staining protocol was tested and refined as a near infrared (NIR) dye rather than a densitometric stain; this proved a broad initial solution to the issue of in-gel detection with sensitivity comparable to SYPRO Ruby while also being easy to use, MS compatible, and very cost effective [99,100,116,118]. Thus, current in-gel detection limits for *intact* proteoforms are in the low femto-to-attomole range, comparable to *peptide* detection in routine shotgun analyses [103].

To dig still deeper into the proteome, it was found that re-imaging 2D gels after excising ~20% of the highest abundance spots of near-saturating signal strength enabled the detection of very low abundance species [102]. These spots, along with areas at the pH extremes and unresolved small species/peptides in the migrating front, can be subjected to a third round of electrophoretic separations (i.e., 3DE), further enhancing the depth of proteome analysis [96,119]. While it was originally thought that one spot on a 2D gel contained perhaps 1–2 protein species, deeper analyses with ever more sensitive mass spectrometers has confirmed that there can be in the range of 200 or more proteoforms in a given spot, depending of course on spot size, density, and thus also quality of resolution [96,98,104,105]. Therefore, optimal in-gel resolution is needed to ensure the best possible assessment and identification of constituent proteoforms by LC/MS/MS [81,96,102]. 

As a mature technology, there also exist variations of 2DE, the most extensively used being two-dimensional *difference* electrophoresis (2D-DIGE). Utilizing the same fundamental methodology as 2DE, 2D-DIGE enables multiple protein extracts to be resolved on the same 2D gel (i.e., multiplexing) [120]. This method is often used to compare distinct samples and involves labeling each with one of two related fluorophores (i.e., Cy2 or Cy3) and a pool of both samples is labelled with a third (Cy5) [121]. In theory, the objective of this multiplexing is to reduce inter-gel variability.

Two labeling methods can be employed in 2D-DIGE: minimal (lysine-reactive) or saturation (cysteine-reactive). The lysine-reactive dyes are hydrophobic and label by covalent modification, leading to the removal of multiple charges from the protein species [95,107]. Unfortunately, this causes a decrease in solubility and can lead to sample loss through precipitation. Since lysine residues make up ~6% of all amino acids in human proteins, a saturation labeling approach cannot be used as it can cause large p*I* and MW shifts [122,123,124]. Thus, with minimal labeling, <5% of each protein species is thought to be labeled (estimated as one lysine residue on one out of twenty protein molecules) [95,107,122,125,126,127]. It has been seen that a single dye molecule (434–464 Da mass addition) per protein species has negligible impact on p*I* and MW of proteins <30 kDa, however proteins of these smaller sizes have poor mobility compared to their unlabeled counterparts, decreasing the quantitative capacity of the approach [95,122,126]. Additionally, these dyes react with thiol-based reductants (i.e., dithiothreitol) [95] and therefore the samples cannot be reduced. However, sample reduction is extremely important for breaking disulfide bridges prior to electrophoresis which helps reduce streaking of spots within 2D gels and the potential identification of ‘false’ proteoforms [128,129,130].

Saturation labeling is said to be somewhat less of a concern with cysteine residues as there are, on average, fewer per protein compared to lysine [122,126]. Cysteine-reactive dyes are fluorophores using maleimide chemistry to label all free cysteine residues. Since the reaction is with the thiol group of cysteine, the sample requires reduction prior to labeling to expose the residues [126,127]. Unfortunately, only ~96% of human canonical proteins possess at least one cysteine residue [131], leaving the remaining canonical sequences unlabeled; an additional proportion may not be labeled if the cysteine is not free to react following reduction.

Similar to issues noted with Isobaric Tags for Relative and Absolute Quantification (iTRAQ) (Section 4.3), it is possible that lower abundance proteoforms are less likely to be labelled, especially if lysine or cysteine residues are blocked by PTM or modifications to neighboring residues [117,126,132]. Lysine-reactive dyes are based on *N*-hydroxy-succinimidyl (NHS) esters which undergo nucleophilic substitution with ε-amine groups of the lysine residues [126]. Importantly, of the 20 amino acids, lysine is one of the most heavily modified [133]. Lysine is often found at many functional sites (e.g., enzyme active sites and interfaces mediating protein–protein interactions) and is frequently covalently modified by acetyl, hydroxyl, propionyl, butyrl, crotonyl, ubiquitnyl, ubiquitinyl-like (SUMOylation, ISGylation, NEDDylation), formyl, malonyl, succinyl, and methyl groups ([124,133,134] and references within [133]). Lysine methylation occurs when up to three methyl groups are transferred to the ε-amine [133]; thus, any proteoforms with these PTM will not be detected with the NHS-CyDye. Notably, the chemical labelling of a proteoform with a CyDye can be considered an adduct and thus, there is also the possibility of the dye disrupting inherent modifications and thus changing proteoforms (although this has yet to be demonstrated).

While cysteine residues are not as prone to PTM as lysine residues, the thiol groups have unique nucleophilic and redox properties that support modifications including oxidation, S-nitrosylation, palmitoylation, prenylation, and Michael addition with oxidized lipid species. Further, while it is rare, cysteine has been seen to be methylated or phosphorylated ([135,136] and references within [135]). Therefore, numerous modifications may block dye binding, further decreasing the quantitative capacities of 2D-DIGE. Again, the reactive dye may also alter existing PTM (e.g., via disruption of thioester bonds). Thus, while an interesting concept, 2D-DIGE does not appear to ensure genuine quantitative assessments of proteomes. Indeed, this appears to be an issue with all covalent labelling-based methods.

Considering the extreme complexity of proteomes, it would thus seem that such targeted methods will not quite work in the manner originally hoped. Noncovalent dyes binding to multiple residues (i.e., SYPRO Ruby and cCBB) thus appear to provide better quantitative detection but it does not appear that a comparable covalent dye has been identified. That is not to say that the binding of noncovalent dyes cannot also be blocked by PTM, but the likelihood of that occurring across the entire surface of a proteoform seems, on average, far less likely.

Thus, while the integrative analytical approach provides high-resolution proteoform separations, there is still room for improvement. In this regard, two principal issues remain with 2DE. First, occasional precipitation in IEF, usually of high abundance species, can interfere with quantitative transfer into the second dimension. This can also confound the identification of genuine protein oligomers as opposed to non-native aggregates formed due to co-precipitation. Although analyzing membrane and soluble proteomes separately [97,102], and supplementing the standard CHAPS detergent with others, in particular a lysolipid [137], seems to help, this issue must be more fully addressed to ensure the best possible proteome analyses.

The second issue concerns the capacity to recover intact protein species embedded in the polyacrylamide matrix; this has been a long-standing challenge in terms of subsequent MS analyses. As the Bis (N,N’-methylene-bis-acrylamide) cross-linked polyacrylamide matrix is insoluble, it is difficult, if not impossible, to quantitatively recover the embedded intact proteoforms; recovery via electroelution is possible but of variable-to-low efficiency, especially for high MW species, leading to a reduced depth of quantitative proteome analysis [138]. Alternatively, the peptides of resolved proteoforms are more routinely released via digestion, most commonly tryptic. While the original concept of a ‘molecular scanner’ [139] might in theory address this concern it would, minimally, still require genuine quantitative recovery (i.e., transfer) of all proteoforms resolved in the 2D gel. Furthermore, while dissolvable polyacrylamide matrices have been known for several decades [140,141], the chemicals needed would likely result in nonspecific and non-native alterations to the proteome.

**Figure 7:**
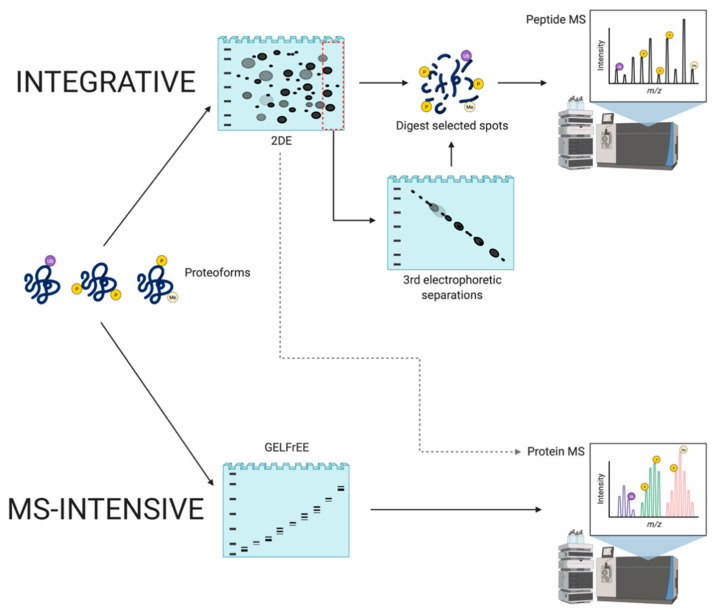
Integrative and MS-intensive proteome analysis. This schematic depicts the workflows of these two top-down approaches. Integrative MS involves the separation of intact protein species via 2DE prior to peptide MS. Additionally, spots of high abundance or areas at the pH extremes and unresolved small peptides in the migrating front can be further subjected to 3rd electrophoretic separations. MS-intensive involves separation of intact protein species, currently mainly via GELFrEE, prior to intact protein MS. Dashed line represents the potential combination of integrative and MS-intensive approaches, which has not yet been pursued.

Another important approach to top-down proteomics that has emerged involves the application of MS to analyze intact proteoforms as opposed to identifying proteolytic peptides [142,143,144]. As this method has not yet been integrated with 2DE/gel-based analyses (aside from Gel-Eluted Liquid Fraction Entrapment Electrophoresis [GELFrEE]—Section 4.2.3), it is considered MS-intensive top-down (Figure 7). The aim is to characterize intact proteoforms (i.e., including PTM) and it thus differs from the integrative approach as it seeks to avoid proteolytic digestion, and thus full sequence analysis occurs in the mass spectrometer using alternate approaches to disrupt the amino acid backbone [142]; these include collision-induced dissociation, higher-energy C-trap dissociation, electron-transfer dissociation, or ultraviolet photodissociation. However, this approach is also not without technical issues.

#### 4.2.3. MS-Intensive

MS-intensive top-down proteomic analyses require the transition of intact proteoforms (i.e., in solution) to positively charged molecular ions (gas-phase). The exact *m*/*z* of the intact proteoforms of interest are then measured [142,143,144]. While this method removes any inference associated with bottom-up peptide MS and provides full sequence coverage of proteoforms, including number, position, and type of PTM on a single polypeptide chain, there remain many technical challenges to be addressed [87,145,146]. First, intact protein MS cannot match the high throughput obtained by peptide MS and is currently unable to handle large-scale analyses [143,147]. Specifically, the inherent difficulty is in producing extensive gas-phase fragmentation of intact proteoforms. Currently, MS technologies are incapable of handling intact proteoforms of larger sizes and mixtures of proteoforms with different physico-chemical properties; this results in loss of certain components or incompatibility of the protein species with MS [78]. Thus, this method can only be consistently applied to species of less than ~30 kDa; there are some reports of select identifications of proteoforms >30 kDa, even up to 104 kDa, although this is completely dependent on the capacity of a few random species to effectively fragment using available technology [18,143,144,145,147,148]. Thus, in studies identifying larger proteoforms (e.g., >100 kDa), these are currently exceptions—the majority of species that can be effectively analyzed are in the low MW range (i.e., 3–30 kDa) and only a very small handful of larger MW species can currently be effectively identified.

To address these limitations, technological innovations have been developed to improve the assessment of high MW biomacromolecules in various types of samples. However, this process becomes increasingly difficult as sample complexity increases because several components can have exceedingly small differences in their *m*/*z*, which makes the analysis of their mass spectrum difficult. Due to the high dynamic range of concentrations in native samples, proteins of higher abundance can suppress the signal of lower abundance proteins (as also seen in 2DE—Section 4.2.2) [96,149]. To address this, a high-resolution mass spectrometer can be used to discern small differences in mass, along with a prefractionation technique to reduce sample complexity and chemical noise. Thus, front-end separation of total protein extracts (i.e., the proteome) is necessary to obtain accurate and reproducible results. GELFrEE has been introduced as a prefractionation step prior to MS-intensive analyses. Quite simply, this is single dimension continuous elution tube gel electrophoresis providing low resolution separation of proteins by MW [109,150]. Such continuous elution gel electrophoresis approaches are well-established, and comparable equipment has been commercially available for more than two decades (i.e., the Prep Cell, Model 491, and BioRad) [151,152] as well as additional designs [153], including simple adaptations to widely used SDS-PAGE gel systems [154]. Such low resolution SDS-PAGE gel-based separations are done prior to the MS-intensive analyses in order to decrease sample complexity. However, there is the issue of possible inconsistency of run times, and this additional up-front gel-based separation step does not correct the current inability of the in-line approach to analyze larger proteoforms but rather fractionates them from the lower MW species that can be analyzed.

In addition to GELFrEE, passive elution of species from SDS-PAGE gels has also been trialed. When comparing the mass spectrums from samples handled with this workflow vs. GELFrEE, they yielded a similar number of proteoform identifications; however, both prefractionation methods still resulted in fewer identifications as the MW of species increased. This can be partially attributed to the decreased recovery rate of high MW proteoforms as the median recovery rate for proteins below 100 kDa was 68%, whereas for those above 100 kDa it was lower (~57%) [155]. Thus, while the MS-intensive approach can indeed analyze some fraction of proteoforms, it is nonetheless also reliant on front-end gel-based separations, raising interesting questions concerning complementarity and integration of approaches to drive the most robust proteome analyses.

Considering the sheer amount of detailed data that the MS-intensive approach can extract per proteoform, one must also understand how the analyses are carried out. The use of a soft ionization technique is paramount as this does not induce dissociation and maintains the integrity of the proteoforms under investigation. Among all available ionization techniques, ESI is the most commonly employed due to its high sensitivity, ease of integration with LC, and its ability to produce multiply charged ions [156,157]. Matrix-assisted laser desorption/ionization (MALDI) is another frequently used soft ionization technique, however, because it predominantly yields singly or doubly charged ions, conventional MALDI is less favorable than ESI for the study of large biomacromolecules [158]. This is a significant disadvantage as multiple charging decreases the *m*/*z* of the ions so they may be analyzed within the ranges of most standard mass spectrometers [156,158]. Recently, the development of small emitter tips has allowed for the use of physiological concentrations of non-volatile salts in ESI systems as the use of smaller droplets reduces salt adduction and improves the resolution of charge-state distributions [159]. While analysis of large proteoforms is possible with ESI on older mass spectrometers, it is difficult as the signal-to-noise ratio (S/N) decreases as a function of increasing MW. As the MW of a species increases, so too does the number of charges it can carry and hence, the number of possible charge states, seen as a peak on a mass spectrum, and can vary depending on a number of factors such as pH, protein conformation, or ambient pressure [160,161]. Each peak is surrounded by a cluster of smaller peaks, the number of which depends on the various combinations of naturally occurring isotopes, further contributing to noise. Indeed, the isotopic effects on S/N are more pronounced at a low MW, whereas charge state effects begin to dominate at a higher MW, however, both need to be considered. Additionally, the effect of chemical noise stemming from various factors such as analyte clustering, multimers, or interfering species, further compounds the arduousness of intact protein detection and analysis. When modelling the decay in S/N against increasing MW with the aforementioned effects considered, there is a pronounced decrease in S/N at a mass of 20–30 kDa. This emphasizes the importance of developing effective separation strategies to remove interfering species, as well as high-resolution mass spectrometers which can distinguish compounds that would otherwise appear as a single peak [161].

Fourier-transform ion cyclotron resonance (FTICR) MS is the gold standard in terms of high-resolution MS and can be coupled with ESI, making it the most valuable instrumentation for intact protein MS-analysis (and by far the most expensive). An FTICR functions by using a Penning trap, which confines ions radially and axially using a magnetic and electric field, respectively. Once excited to their resonant cyclotron frequencies, the ions travel near detection electrodes on which they induce an image current. The signal is then converted to the frequency domain via Fourier transform, from which *m*/*z* can be calculated [162,163]. Given that FTICR-MS performance metrics are directly related to the field strength of their magnets, the new 21T FTICR-MS offers the highest resolving power and mass accuracy available (*m*/Δ*m*_50%_ > 2,700,000 at 400 *m*/*z* and 80 ppb, respectively) [162,164]. However, the increase in field strength has not yielded an expected proportional increase in mass resolving power when compared to the 18T FTICR-MS. Currently, the potential of the 21T FTICR-MS is restricted by the inability to produce a sufficient vacuum near the ICR cell because the mass resolving power in an FTICR-MS is equal to the frequency resolving power, which depends on the acquisition time, assuming the collision-free motion of an excited ion. A greater acquisition time allows for more data points to be collected, which results in a greater resolution; however, collisions between the analytes and background gas molecules results in signal decay and decreased resolving power. A high vacuum in the ICR cell is therefore required for optimal function [162,165,166]. Recently, a concept was developed for a modified dynamically harmonized cell with a new “zigzag” ion trap configuration, which can improve the vacuum by decreasing the surface area of the cell and incorporating the vacuum tube directly into the working region of the cell however, this has not yet been implemented in an FTICR-MS so the practicality of this new cell remains to be demonstrated [167]. Additional disadvantages of the FTICR include its extremely high upfront and maintenance cost due to the requirement of cryogenic cooling for its magnets [168].

An alternative to FTICR is the Orbitrap, another Fourier transform MS, which provides comparable resolution and accuracy. In contrast to the FTICR, in the Orbitrap, ions are trapped only using an electrostatic field, rather than a magnetic field [169]. The motion of ions differs from the FTICR in that they oscillate along and rotate around the central electrode. Due to the electrostatic field, the kinetic energy of the ions within an orbitrap is typically greater because it is dependent on the force exerted by the electric field. Conversely, in an FTICR the kinetic energy decreases as (*m*/*z*)^−1^, allowing for longer acquisition times and hence, greater resolution [170,171,172]. Consequently, Orbitraps experience a faster signal decay as a function of increasing MW due to greater intermolecular collisions, limiting its routine analysis of intact proteins to those under 30 kDa. However, by modifying the Orbitrap, researchers have attempted to improve its capabilities in niche circumstances [170,171]. For instance, it has been demonstrated that trapping ions in the higher energy collisional dissociation cell and replacing the nitrogen environment with helium can reduce the number of ion collisions, resulting in greater resolution and the mass determination of a 148.7 kDa IgG1 antibody [173]. While interesting, most such analyses of intact high MW molecules have been conducted under ideal conditions, using highly purified, commercially supplied proteins, rather than extracted native proteomes which carry a significant degree of chemical noise.

An interesting recent development is individual ion MS (I^2^MS), which demonstrates great potential for the characterization of complex proteoform mixtures. In I^2^MS, each individual ion is analyzed independently, and the image current induced is plotted as a linear function of acquisition time, the slope of which is proportional to its charge. Using this function, each ion is assigned a charge which is used with *m*/*z* to produce a true mass spectrum [144,174,175,176]. In a recent study using I^2^MS, 550 proteoforms were identified, along with a group of unidentified proteoforms resolved between 20–25 kDa, from HEK293T cell lysate fractionated by GELFrEE [176]. Furthermore, two engineered virus-like particles (VLPs) produced in *E. coli* and carrying DNA and RNA were analyzed using I^2^MS yielding high-resolution mass distributions with masses of 990 ± 16 kDa and 3190 ± 38 kDa [144,176]. Nonetheless, while quite promising, I^2^MS is also not without limitations. Given that multiple ions are analyzed in one acquisition, more than one ion may produce the same frequency corresponding to the same *m*/*z,* which would lead to false charge assignments. Furthermore, while 550 proteoforms were identified, it was not directly reported how many canonical proteins this represented. Additionally, the variability in the linear function used to assign charges decreases proportionally to (ion survival time)^1/2^ which, as discussed above, is a limitation of the Orbitrap. In an effort to rectify this, voltage has been decreased to lower the kinetic energy of the ions, however, the proteins analyzed were either small enough (<30 kDa) or too large (0.99 and 3.19 MDa) to be significantly impacted by inter-molecular collisions [176].

As the MS-intensive approach can currently analyze primarily a lower MW sub-set of species, it is not yet capable of large-scale investigations of ‘complete’ proteomes [18,143,144,145,147,148]. In saying that, this method can comprehensively characterize proteoforms, strongly suggesting that MS-intensive top-down will be an extremely powerful tool in the future, when current technical limitations are overcome, enabling it to be more broadly applicable, routine, and far more cost-effective. It would be ideal to pair the current MS-intensive and integrative methods, although this will not be possible without further refining ***both*** methods. Clearly, this is an extremely critical area for development.

#### 4.2.4. So, What Does Top-Down Really Mean?

The development of proteomics clearly began with a top-down approach, 2DE. Until the advent of the MS-intensive approach, 2DE was essentially the only method to resolve intact proteoforms and ensure the best subsequent analyses to identify species [29,104,177]: it arguably still is. The evolution of this method has resulted in some debate in the field as to which analytical approach is truly ‘top-down’. Since proteomics is analytical chemistry and both methods first resolve intact proteoforms, then, regardless of how proteoforms are eventually identified (i.e., fragmented before or after entering the mass spectrometer, or via Western blotting), they are both top-down methods. 

To put this into perspective, when 2DE ‘was’ proteomics, and Western blotting was the primary means to identify specific proteins (along with occasional analyses by Edman degradation), it was the recognized top-down analysis. As analytical instrumentation progressed, as it does, different forms of MS were progressively adopted to complement the resolution achieved with 2DE and to identify canonical protein sequences; doing so with an alternate, low resolution up-front gel electrophoresis method (e.g., SDS-PAGE/GELFrEE) does not change that approach. Should another, ‘better’ instrument/methodology arrive in the future for proteome analysis, would the MS-intensive approach then cease to be top-down? 

Therefore, instrument-centric definitions are somewhat personal adoptions whereas general systematic approaches are what should be described by the terminology. Accordingly, the integrative and MS-intensive approaches, from an analytical chemistry perspective, both provide top-down analyses. We will not even consider the term middle-down, which is apparently used in some bottom-up and modified MS-intensive circles to describe a protocol that uses limited proteolysis/larger sizes of peptides to enable analyses. However, it has also been used to describe a number of different approaches in the field, confusing matters to the point that its use becomes essentially meaningless [28,68,178,179]. Again, here is another area in which consensus would be useful and important, to move the field forward with a unified terminology and understanding of the genuine pros and cons of available analytical approaches [27,28,29,51].

### 4.3. Additional Analytical Variations on Peptide MS Analyses

Alternate analytical approaches that seek to enable quantitative proteome assessments include mainly variants of peptide MS such as iTRAQ. This multiplex approach seeks to identify changes in the abundance of canonical protein sequences, simultaneously, in up to eight biological samples, using isobaric tags to label the N termini and lysine side chains of peptides, ostensibly for either relative or absolute quantification [180,181,182,183,184,185]. This method is claimed to have high sensitivity and reproducibility, although it has recognized biases and underestimation issues [182,183,185]. Specifically, when two or more precursor ions with similar *m*/*z* and retention times are selected in the same fragmentation window and are sequenced and quantified together, both peptides contribute to one MS/MS signal. Thus, both are sequenced and quantified at the same time even though they are two separate species [183]. One of the crucial issues with labeling methods such as iTRAQ is that not all peptides have lysine side chains and, therefore, not all peptides will be labeled, thus yielding results that cannot be considered quantitative. Furthermore, any such labelling reactions, with reactive fluorescent groups or isotopes, will not be 100% efficient and hyperabundant species will dominate the reaction. Thus, any quantification must be thoroughly validated by orthogonal methods. Only stable isotope labeling by amino acids in cell culture (SILAC; Section 5.3.1) is likely to yield absolute quantification but is clearly not applicable to most sample types analyzed in proteomic studies (e.g., tissue and fluids); the exception may be *Drosophila* embryos fed labelled *S. cerevisiae* [186,187].

While peptide MS largely focuses on identifying amino acid sequences, it is possible to identify PTM, although it demands substantial additional work and time, and significant complications can arise. Foremost, these approaches generally require enrichment techniques—increasing the concentration of select proteins or peptides to improve their downstream analysis—and these selection techniques differ (including in quality and rigor) for every specific PTM that is to be analyzed [188,189,190]. Additional issues may arise in the MS analysis. For example, while identification is possible, phosphopeptides are not ionized and fragmented as efficiently as unmodified peptides making MS identification more difficult [190,191,192]; this is further complicated by the potential presence of ‘non-standard’ phosphorylation of histidine, arginine, and lysine. Another PTM, ubiquitination, results in a mass shift of 114.043 Da. Unfortunately, other events (e.g., a cleavage between a lysine-asparagine motif on other peptides) have been seen to cause an identical mass shift making it difficult to distinguish them from ubiquitination [190,193]. Different enrichment techniques also exist for other PTM (e.g., glycosylation, acetylation, methylation, and cysteine redox modifications), although a separate sample aliquot is needed for each identification and once used to identify one PTM, it cannot be re-used to identify another type of modification: clearly this may be an issue depending on amount of sample available. The question also arises as to which PTM should be analyzed, as each choice thus introduces added analytical bias while still not ensuring definitive identification of proteoforms since the starting material is a proteolytic digest of the proteome. Furthermore, as each PTM requires a separate analysis in addition to the original shotgun process, this essentially turns a high throughput discovery approach into a targeted search for specific alterations based on an assumption of what the important PTM might be. Again, it would seem impossible to do this systematically or exhaustively and thus, impossible to effectively analyze a native proteome (i.e., the full spectrum of constituent proteoforms). However, if there are specific PTM of interest (i.e., known or strongly suspected to be involved in a biological process of interest), then a targeted approach for specific species can be used—with the usual caveat of the need for attention to weaknesses of the methods in order to capitalize best on the strengths.

## 5. Targeted Proteomics

A discovery approach can potentially generate 100s–1000s of hits, necessitating a targeted approach to validate the identified protein species if they are to later be assessed on a larger and/or more routine scale. Targeted proteomic workflows thus involve the detection of a canonical protein or proteoform of interest, hopefully with high sensitivity, selectivity, quantitative accuracy, and reproducibility [66,67]. This approach is essentially the bridge that connects discovery proteomics to the validation of biomarkers, potential targets for drug development, and other research efforts. Unfortunately, the quality of this bridge is heavily dependent on a number of factors and criteria [194,195]. There is a bottleneck as the techniques for targeted proteomic studies cannot keep up with the number of reasonably strong hits being made by discovery approaches. This has resulted in a seemingly perpetual gap in the identification of protein targets vs. the testing/validation of potential biomarkers and drug targets. It is most often seen that only a very small number of protein/proteoform candidates (most often those that show the most significant differences in abundance between two or more conditions) are further tested using a targeted approach as current methods can be extremely time consuming and expensive, obviating the capacity to separately test each potential candidate found in discovery studies [195,196]. Furthermore, quantitative rigor varies between methods and how they are applied, again emphasizing the need for consensus to address inter-lab variability or outright irreproducibility of findings. Thus, similar to discovery proteomics, there are numerous approaches used for targeted proteomic analyses although, again, each has its own strengths and weaknesses. Initially we will address those more common methods that utilize antibodies for detection of the target species.

### 5.1. Antibodies

Immunoassays are likely the most widely used method for targeted proteomics, utilizing antigen-antibody interactions for detection and quantification of the target species. Before discussing the numerous immunoassay methods that exist, it is important to delve into the advantages, as well as the limitations, of the main component used.

There are two main types of antibodies: monoclonal (mAb) and polyclonal (pAb). The former is monospecific, recognizing only a single epitope per antigen; pAb are heterogenous, each antibody component in the mix recognizes a different epitope on the same antigen. mAb are most useful for their strict specificity, and thus in evaluating changes in molecular conformation, protein–protein interactions, and PTM. However, this means that slight changes in the epitope (e.g., genetic polymorphism, untargeted PTM, and denaturation) can affect the binding of the mAb. This can be addressed by pooling multiple mAb of desired specificities, although this can be difficult, expensive, and time-consuming. Aside from epitope specificity, generally the biggest advantages of mAb, compared to pAb, is their high concentration, purity, and reproducibility, which arise from their capacity to be generated from a constant and renewable source. An additional antibody type that is easily sequenced and resynthesized are nanobodies. These monoclonal-like antibodies are devoid of light chains making them small, with high thermostability, superior solubility, and cost effectiveness [197,198,199]. Nonetheless, in some instances the monospecificity of mAb is considered an issue and pAb are preferred [200,201,202]. 

pAb are more stable over a broad range of pH and salt concentrations, often enabling their use under a variety of experimental conditions. Unfortunately, they are non-renewable, and their avidity is at risk of changing as they are harvested over time, and quantity of pAb obtained is limited by the size and lifespan of the host animal [19,200,202]. Additionally, pAb recognize multiple epitopes per antigen thus, if one or more of the clones in the pAb mix recognizes a highly conserved protein ‘domain’ (e.g., a calcium binding domain)—which is found on numerous unrelated proteins/proteoforms—then it can lead to false identifications.

Overall, antibodies do not exist for all proteins, and vary widely in quality as most that are commercially available often seem to be subjected to limited validation [203,204,205]. Critically, there are even fewer proteoform-selective antibodies available. If an antibody does not perform as expected, then alternative antibodies with better performance or an antibody-independent approach must be considered which, unfortunately, is costly and time-consuming [19]. Thus, as with all methods, there are clearly pros and cons to be considered in using antibodies in proteomic analyses (although much can be achieved with rigorous controls and optimization; see below). To reduce interference commonly seen with full-size antibodies, Fab fragments can be used; removing the Fc fragment from the antibody provides for smaller binding components which improve binding capacity and can thus improve assay sensitivity [206,207].

**Figure 8:**
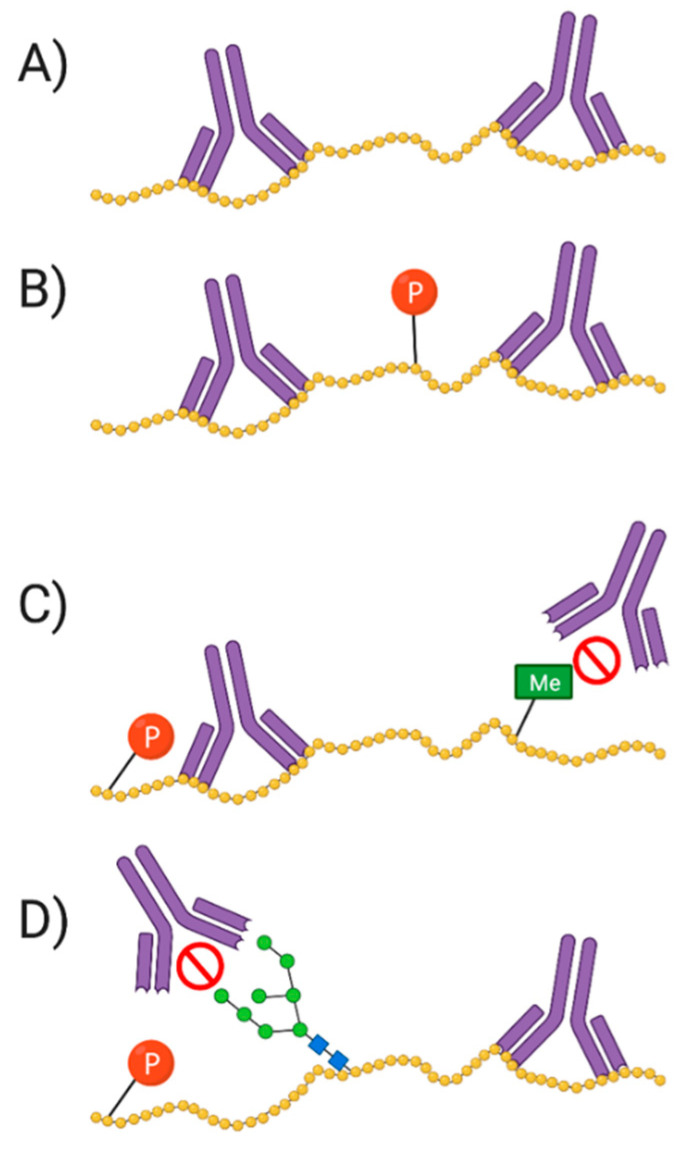
Antibodies and proteoforms. As antibodies are mainly raised to identify amino acid epitopes, it is possible that a PTM at, or near, the epitope will interfere with binding of the antibody. This may prevent the detection of the target. (**A**) Antibody binding without any interference; (**B**) Antibody binding without phosphate group interfering; (**C**) Antibody binding blocked by methyl group; (**D**) phosphate and sugar group adjacent to epitope affect/block antibody binding.

Regarding identification of proteoforms, antibodies are generally raised to identify amino acid epitopes on canonical protein sequences; thus, a PTM (e.g., methyl, phosphate, and sugar group) at, or neighboring, the epitope will likely block binding of the antibody, thus preventing detection of the target or at least one or more related proteoforms (Figure 8) [208,209]. In contrast, while there exist antibodies that broadly recognize PTM (e.g., phosphotyrosine), these are generally of poor specificity and selectivity. For the antibody to effectively identify a specific proteoform, it must recognize both a specific PTM and the sequence surrounding it [202,208]. This can be challenging if the same PTM is present on more than one sequence of the amino acid backbone. Using two different antibodies—to the specific sequence and to the PTM—can often help address such detection issues. Notably, 2DE offers significant advantages in alleviating some of these issues since proteoforms have already been resolved prior to detection and thus a single antibody to an unmodified backbone epitope is often sufficient, provided all other control and optimizations steps have also been taken into account (see Western blotting, Section 5.2).

### 5.2. Immunoassays

Of all the targeted proteomic methods that exist, immuno- or Western blotting is most commonly used [210,211]. Western blots are effective for small-scale protein analyses and, at least in theory, are relatively simple and cost-effective, and produce data that is easily interpreted [19,212]. While the original goal of the Western blot was to provide a yes or no answer about the presence of a target protein, refinements over the last several decades have enabled sensitive and reproducible quantification of a given target in a native sample extract [64,65,213]; however, this can only be done when the complete sample is represented (i.e., no proteins/proteoforms are (non)specifically removed due to prior use of fractionation techniques) [214]. Unfortunately, Western blotting lacks the throughput to routinely quantify large fractions of a proteome and quantification depends heavily on the quality of the techniques and antibodies used [19,64,84,205]. There are many factors that can affect the reproducibility and quantitative capacity including large protein loads (i.e., signal saturation) and failure to optimize buffers, blocking reagents, or transfer conditions; the presence of lipids or carbohydrates can also interfere with resolution and detection [19,205]. Additionally, at the initial sample preparation stage, improper handling, poor homogenization, as well as inadequate detergents and protease inhibitors can lead to decreased reproducibility and sensitivity [215].

Regarding technique, transfer method/conditions, gel composition, and type of blotting membrane can all substantially affect the quantitative efficiency of protein transfer from the gel to the blotting membrane [19,216]. Two commonly used transfer methods are wet (complete immersion of a gel-membrane sandwich in buffer) or semi-dry (gel-membrane sandwich is placed between absorbent paper soaked in transfer buffer). Wet transfer has high efficiency but takes more time whereas semi-dry is convenient and saves time, although this is often at the expense of transfer quality; specifically, higher MW proteins may not transfer as effectively as they do with a wet transfer [19,216,217]. Notably, a more complete and higher quality transfer is seen when thinner gels are used but there is the added risk of gel cracking/ripping during handling [19]. 

Transfer conditions (i.e., current, voltage, and buffer) also play key roles in transfer efficiency. Similar to the concept of protein separation with SDS-PAGE, low MW species generally transfer faster than those of higher MW. Therefore, under conditions optimized for transfer of lower MW species, larger proteins have low transfer efficiency and under conditions optimized for high MW proteins, those of lower MW can be driven completely through the blotting membrane [64,216]. This can be avoided with vacuum-assisted solvent flow (transfer of proteins from gel to membrane using suction power); however, as stated in Section 4.2.2, this is difficult with acrylamide gels and if transfer is extended longer than 45 min, the gel is at risk of drying out [216,218]. 

Furthermore, some PTM (e.g., glycosylation) can markedly affect transfer efficiency, so conditions must always be optimized for species of interest [216,219,220]. The practice of diluting the primary antibody to only detect a single band has been used by some as a proxy for appropriate optimization steps but can prove to be quite misleading. Additional bands may be indictive of notable proteoforms and/or modification (e.g., cleavage) due to suboptimal handling rather than be ‘spurious’ (e.g., the result of nonspecific antibody binding) [19]. The presence of proteoforms and aggregates/multimers in different bands can be easily confirmed via MS/MS, ensuring the most thorough and quantitative Western blot analyses. Thus, a well validated primary antibody is critical to success, as is a high-resolution separation of species present in the sample; this also emphasizes the risk of interpreting dot blots beyond simply indicating that the antibody being used is binding to something in the sample (which may or may not include your species of interest). 

Overall, while it is relatively simple to detect proteins blotted from gels, doing so reproducibly and with quantitative rigor requires more effort than is commonly seen in the literature. Simply, one size does not fit all, and the common assumption that the ‘standard’ protocol used in a given lab will work effectively for every sample or species is false. Select optimizations and standards for quantitative calibration may well be needed for each distinct species under investigation. Routine controls should always include (i) ensuring uniform total protein loads per sample (rather than relying on the misleading and outdated use of ‘housekeeping’ proteins for normalization) and (ii) quantitative assessment of transfer efficiency using the highest sensitivity in-gel detection available. This will minimally enable quantification relative to the control samples. 

Regarding detection methods, the two most known are chemiluminescence and fluorescence. Colorimetric methods do exist but their performance varies based on purity of substrate and buffer components [221]. Chemiluminescence is used most widely and involves an enzyme-substrate reaction to generate light. The emitted light then decays to ground state and the signal fades quickly, often within a very brief time [221,222]. This degradation is quite disadvantageous as the signal is not consistent, and once it is terminated, the ability to retrieve any additional quantitative data in the future becomes more difficult. An improved method utilizes chemifluorescence, which yields stronger and more stable signals, enabling more sensitive detection via signal integration over time [64,65,221,223]. Additionally, antibodies can be tagged with fluorophores, some detectable in the infrared spectrum. NIR reduces the risk of and thus interference by autofluorescence (natural emission of light) [223]. Since the excitation/emission capacity of these fluorophores does not diminish over time, they enable extended signal integration and thus greater sensitivity of detection. Furthermore, using different fluorophores enables multiplex Western blot assays [224].

Another commonly used method for targeted protein detection is an enzyme-linked immunosorbent assay (ELISA). This method is similar to Western blotting in that it requires an antibody to identify the target. However, ELISA differs as its visual readout is in a 96-well plate as opposed to a blotted SDS-PAGE gel and thus there is no resolution of species (e.g., always leading to the same potential concerns as with a dot blot). The type of catalytic label used can produce different methods of visual detection similar to that of Western blotting (i.e., colorimetric, chemiluminescent, and fluorescent) [225,226]. While ELISAs are often thought of as reasonably fast with a high degree of sensitivity, specificity, and reproducibility, they unfortunately share a critical limitation with Western blots: whether or not the technique is optimized to achieve maximal assay quality, the method is largely dependent on the quality of the antibodies used [194,226]. Unfortunately, these are largely considered proprietary by the commercial firms producing most of the ELISA kits used in current research and, certainly in our experience when purchasing kits, it is impossible to also get samples of the antibodies to verify their target specificity in the samples being analyzed. Like dot-blots, one is assuming that only the target of interest is contributing to the signal, with the understanding that the signal likely represents a host of proteoforms.

Similar to ELISAs are single molecule array assays (SiMoA). This method uses paramagnetic beads that are coupled with biotinylated detection antibodies, streptavidin-labeled enzyme, and the target molecules (proteins) to form immunocomplexes. These beads are then added to a microwell array and a non-fluorescent resorufin-β-_D_-galactopryanoside (RDG) is added. RDG is converted to a fluorescent product when it reacts with the streptavidin-labeled enzyme. The wells in the plates used for SiMoA allow one type of labeled bead per well (i.e., targeting at a single canonical protein); this ensures that the ratio of active beads to number of beads located in the wells is directly correlated to the concentration of the target protein in the sample being examined [227,228,229,230]. The benefit of this assay format is increased sensitivity and ability to detect sub-femtomolar concentrations of the target [230,231] although, it is quite expensive and has similar disadvantages to an ELISA, in that output only represents total abundance of a canonical protein.

Although quite different from the other methods discussed, another antibody-based approach of note is immunohistochemistry (IHC), which is used to detect target in fixed cells and tissue slices [232,233]. This is particularly useful for localizing target species of interest, although quantification is limited to relative comparisons between samples. Nonetheless, much work has been done to improve quantitative assessments. In typical IHC staining, active immunocomplexes produce a broad colorimetric response however, it makes quantitative observation difficult [234]. An alternative method, quantitative IHC, uses an additional enzymatic amplification which converts the antibody/antigen complexes into defined dots, allowing for counting. Prior to labelling, a pre-determined fraction of secondary antibodies is labeled and thus the ratio between number of labeled sites and number of labeled antibodies can be used as a direct correlation [235]. IHC differs from Western blots, ELISAs, and SiMoAs as it allows visualization of cellular components, can provide morphological information, and thus determines target localization within a cell or tissue. Unfortunately, the IHC protocol is somewhat long and detailed, and thus throughput can be quite limited. Furthermore, as all of these methods use antibodies there is still the risk of non-specific interactions as well as the many limitations discussed earlier. While these methods have been invaluable, they clearly also have limitations. To some extent, recent rapid developments in MS provide some help or alternate approaches in this regard. 

### 5.3. Mass Spectromtery

#### 5.3.1. Label-Based

As an alternative to immunoassay approaches, MS can be used to identify canonical proteins in a targeted approach either with a label-based or label-free method (these are generally discovery-based methods but can be modified for targeted use). The former involves labelling the peptides in a sample digest, prior to shotgun analysis, using different reagents that are chemically identical but differ in their isotopic composition [66,236]. The peptides can undergo chemical, metabolic, or enzymatic labeling—each again with distinct advantages and disadvantages [66,236,237].

The most popular method, metabolic labeling (i.e., SILAC), involves the addition of a stable isotope label to growth media, enabling its incorporation into metabolically active cells [238,239,240]. As this method does not target functional groups and the isotopic label is introduced prior to protein extractions, each newly synthesized protein is labelled efficiently compared to other labelling methods [239]. While the only truly absolute quantitative method, unfortunately, it is limited to cell cultures and not applicable to the vast bulk of proteomic studies that focus on tissues or biofluids [241]. Furthermore, SILAC introduces an additional shift in the isotopic envelope of the mass spectrum that may result in peak overlap which, coupled with the decreased likelihood of complete labelling as a function of increasing protein size, further complicates data analysis of larger proteins [242]. 

Chemical labeling (isotope-coded affinity tagging (ICAT), isotope-coded protein labeling (ICPL), tandem mass tags (TMT), and iTRAQ [Section 4.3]) introduce isotopic or isobaric labels at the protein/peptide level following protein extraction and can be used to tag numerous different types of samples [238,240,243,244,245]. Since the labels are introduced following protein extraction, they must target certain functional groups. ICAT favors cysteine-containing proteins, TMT favors NHS ester-based reactive groups, and ICPL and iTRAQ favor primary amines (i.e., N-terminus and lysine side chains). This is a drawback as not all proteins contain cysteine and/or lysine; thus, unlabeled peptides cannot be used for quantification [239,240]. Furthermore, these methods require additional steps for labelling and protein/peptide recovery in the analytical workflow, and the commercially available labelling reagents are expensive [246]. 

Enzymatic labeling involves the addition or removal of water using ^18^O tracers which results in a 4 Da mass shift between the same peptides in two different samples being compared. The main advantage of this method is that it does not target specific amino acids or require added enrichment steps. Additionally, this method is simple, low-cost, and applicable to all types of samples; its main limitations are seen to be incomplete labeling and the lack of capacity for multiplexing [239,240,241].

#### 5.3.2. Label-Free

**Figure 9:**
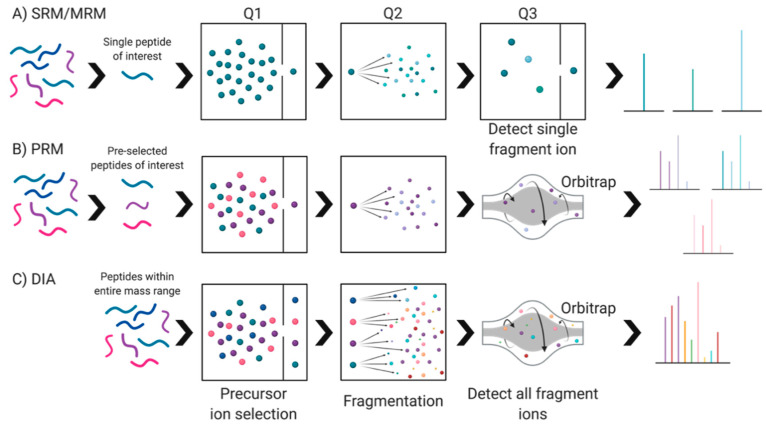
MS-based targeted proteomics. Shown are the different acquisition modes commonly used for targeted detection of protein species with MS. (**A**) SRM—quantifies specific, predetermined ions from peptide of interest; (**B**) PRM—simultaneously analyzes all fragment ions of the pre-selected peptides of interest; (**C**) DIA—analyzes all peptide mass ranges within the window without pre-selection.

In contrast, the label-free method is straightforward, cost-effective, and requires minimal sample manipulation [75,241,247,248], measuring peptides by ion intensity or spectral counting, and is often used to compare two or more conditions (Figure 9) [237,247,249]. Label-free uses the acquired spectra of a given peptide as a proxy for the relative amount of the corresponding canonical protein in a given sample. Again, as a shotgun approach, this assumes all peptides are from intact canonical proteins and may or may not work well with different related proteoforms depending on the nature of the modifications. Thus, this method also tends to lack accuracy, precision, and reproducibility as it does not compare peptides to an internal, chemically identical standard enriched with a stable isotope [75,177,237,250]; that said, this is an area of ongoing refinement [251]. Thus, most simply, label-free provides deeper coverage of the canonical proteome, while label-based methods in theory provide better quantification [177]. Integration with 2DE for front-end proteoform resolution would likely enhance the specificity and quantitative rigor of both approaches [29].

Currently, one of the most used methods for quantification of a given canonical protein by MS is Selective Reaction Monitoring (SRM, and the related Multiple Reaction Monitoring [MRM]) [21,79]. SRM is carried out using a triple quadrupole mass spectrometer, sequentially monitoring for fragment ions from the same peptide between two levels of selection and isolating predefined precursor and fragment ions [67,89]. As this method requires that an assay be developed for each target protein and quantifies specific, predetermined ions, it is somewhat similar to Western blotting, although SRM seems to be somewhat superior with regard to data quality and performance characteristics, provided consistent instrumentation and protocols are used [252]. However, although SRM provides high sensitivity and quantitative accuracy (in terms of theoretically intact canonical proteins), it lacks throughput compared to other methods (i.e., DIA and parallel reaction monitoring [PRM]) as it sequentially samples only one fragment ion at a time [67,253].

In contrast, PRM produces full MS/MS spectra for each precursor and simultaneously analyzes all fragment ions of the pre-selected peptides of interest; SRM only monitors the predefined product ions [67,89,254]. This is advantageous as it provides the flexibility to select fragment ions following data acquisition [67]. Thus, PRM provides higher selectivity, dynamic range, and S/N compared to SRM. Although seemingly not used in any effort to assess specific proteoforms, both SRM and PRM have high sensitivity, specificity, and reproducibility, but they lack multiplexing capabilities. To overcome this limitation, DIA-based targeted quantification may be used.

DIA (Section 4.1) is primarily a discovery-based approach but can also be used as a targeted method. This approach is aimed at utilizing the full capabilities of mass spectrometers to maximize MS acquisition time and to address the need to expand the detectable dynamic range, lower the limit of detection, and improve on the overall confidence of peptide identifications and relative quantification measurements. However, with this multiplexing ability, it is resource demanding and has somewhat decreased sensitivity, specificity, and reproducibility [89].

Like all methods, each of these three label-free methods has its own unique advantages and disadvantages, with some capability sacrificed in each method. Most notable perhaps is their essentially exclusive use in proteogenomic analyses rather than addressing the need to assess proteoforms. Overall, the ‘ideal’ method would provide high throughput, multiplexing capabilities, and high sensitivity, specificity, and reproducibility, and retain these qualities even for the analysis of specific, even closely related proteoforms (i.e., be able to differentiate between them, which is likely impossible at the peptide level). While the instrumentation has seen a steady series of significant improvements over the last two decades, technical limitations may well stand in the way of further major advances. Regardless, any such refinements will also come with a hefty price. For now, we can either wait for this ideal instrument and associated methodologies that will provide thorough MS-intensive analyses of intact proteoforms and thus full proteomes or use the best available technology to address pressing research concerns in medicine, environmental, agricultural, and other areas. This is by no means a suggestion that technological refinement and optimization should not continue, for *all* available approaches to proteome analysis. Rather, it is a comment on better capitalizing on what is available, in parallel with improvements to instrumentation.

## 6. What Next?

Proteomes are of proteoforms, not canonical proteins. There is no one-size-fits-all method for every type of proteoform or proteomic study—discovery or targeted. We hopefully adapt to capitalize on the strengths and limit the impact of the weaknesses in each case. Currently, no single approach is close to reaching the goal of identifying and characterizing ***all*** proteoforms in a proteome; for that matter, we cannot even guarantee full proteome extraction from any given sample. Does this mean we cannot move proteomic research forward without a significant disruptive change? While such sudden advancements can revolutionize a field, they are not the only means of carrying out the best possible proteome analyses. Thus, stringent optimization of and consensus on available methods and criteria for data quality, would be the most obvious and straightforward approach to effectively addressing the inherent complexity of proteomes. Indeed, openly accepting the complexity, by consistently addressing proteoforms as the critical species that must be resolved and identified, would seem the first step. Simply, continuing a purely proteo-genomic/-transcriptomic approach to cataloging amino acid sequences will add a few more entries to databases, but will not effectively address proteome complexity or thus enhance our understanding of molecular mechanisms or identify selective therapeutic targets and biomarkers. We need to integrate and capitalize on what works best—*but use it better*—and put effort into consistently and critically improving approaches to proteoform resolution and assessment to reach the goal of routine, full proteome analyses. Obviously, it is unrealistic to expect such routine, deep proteome analyses within even the next decade considering it took concerted international effort coordinated by HUPO over >10 years to reach the current milestone of 90.4% coverage of the conservatively defined genome at the level of amino acid sequences (i.e., Proteome-lite) [70,71]. Since things are more complex than originally defined, we need to have a firm grasp on the genome if we hope to effectively define a proteome with myriad potential proteoforms that also vary temporally [56]. Importantly, this recent HUPO announcement [70,71] is a new starting point, and a new opportunity for the next generations of researchers to take a bold new perspective on what needs to be done and thus how best to rigorously address the complexity of proteomes.

With these caveats in mind, in terms of a complementary and broadly applicable approach, 2DE/3DE (with a host of well-established modifications and variations to enhance resolution and detection sensitivity/selectivity) coupled with LC/MS/MS (also with established variations to optimize both sample and data analyses) seems to be the only current approach that can effectively resolve, identify, and quantify the largest number of *proteoforms* in a given sample. Essentially this is about escaping technique- or technology-centric biases and integrating existing top-down and bottom-up approaches: capitalizing on existing strengths and minimizing different technical limitations in the process. In saying this, it is also important to note that even though they have undergone numerous enhancements, the core methods can always be further improved. Many critical questions still need to be addressed. Can we further improve protein extraction with alternative detergents and methods? Can we further improve resolution in IEF? Can we further automate any steps within the full analytical process? These are but a few examples deserving of more robust consideration rather than continuing to do things ‘because that is how they have always been done and others do it similarly as well’. If the latter common rationale were actually true—that is, things are *not* in fact done identically between labs, even apparently using the same protocol and instrumentation—there would not exist the current level of inter-lab variation and irreproducibility of findings [255]. The real issue seems to be whether the field seeks to continue its now almost blind commitment to the speed of analyses or whether quality, depth, and quantification in proteoform analysis become recognized as the critical objectives, as will be absolutely necessary with respect to proteome complexity. It is thus time to look forward and fully embrace the genuine complexity of proteomes, and what it will mean to effectively analyze them, particularly in any sort of routine manner. This will require an innovative and transdisciplinary mindset to create an integrative, state-of-the-art *proteomics* that (re)defines the discipline. This is the next generation approach that will future-proof the field and enable nimble integration of advances in both sample and data analysis.

Thus, while MS-intensive analysis now seems to be the most promising long-term venture in top-down proteomics, it currently cannot deliver the depth of proteome coverage provided by the integrative approach. Strangely, it seems a genuine interface of the two approaches (i.e., 2DE and MS-intensive) has never even been tested, although some have reported limited success with proteoform identification following passive elution from SDS-PAGE gels [155], certainly suggesting that this could be quite informative. Furthermore, as we work toward this goal of mapping the proteome [70], all available approaches/methodologies/subdisciplines need to work in unison. As but one example, with the current refined state of 2DE, every spot in the gel essentially becomes a mini bottom-up experiment as 10 s–100 s of proteoforms (or more) can be found in a single spot [98,104,256]. Thus, the best such analyses depend not only on the highest quality 2D/3D gels, but also the highest resolution MS coupled with the best possible data analysis/database interrogations to ensure solid proteoform identifications and, hopefully, deeper analyses to fully characterize the inherent PTM. What if each such spot could be quantitatively eluted and the intact proteoforms fully assessed using ongoing refinements in MS-intensive analyses? Given the current status of technology in the field, it is actually somewhat surprising that the major MS instrumentation firms—that do much to define and manage the direction of current proteomic analyses—do not offer a refined front-end 2DE suite to complement their latest LC/MS instrumentation packages. That would define a very real interest in deep proteome analysis rather than a continuing primary focus on proteogenomics, which will simply not effectively address the complexity of proteomes. Analytical rigor in addressing proteoforms is needed, not just new instruments.

Unfortunately, there has come to be an almost wholesale emphasis on the speed of analysis—we must analyze proteomes at the same rate we analyze genomes—yet apparently ignore all the issues that have arisen and are still appearing with those approaches despite the astounding technologies that have been developed to address genome sequencing demands. In this regard, rather than almost exclusively focusing on ‘fast’ analyses of canonical protein sequences, it seems likely that funding agencies are also expecting to see more analytical depth from investments already made for instrumentation. Realistically however, there is currently no panacea to either deep genome or deep proteome analysis. Yet we continue to claim otherwise rather than appreciating the inherent complexities of the systems—which is where the actual answers to our most critical research questions lie. Those who are seemingly convinced that we will sort this all out ‘tomorrow’ undoubtedly also have a new technology they want to sell you. Noting these issues and that the proteome is so much more complex than the genome, we perhaps need to come to yet another firm consensus as a field by asking a critical question: what is more important, speed or the actual quality of the analysis and resulting data? Then, can those data be turned into knowledge?

We need to take the time to refine and optimize these methods, and present trials/papers exactly as such, rather than claiming they are full solutions and ready to provide a ‘breakthrough’. Some of this may be attributed to publication hype by some journals, but rather than moving the field forward, it is muddying the waters. To achieve any type of Systems Biology, we need to stop ignoring the flaws in our respective approaches/methods. We are at a point where each one of these methods is like a separate piece of a car. If we try to drive with just one piece (i.e., engine) we will not get anywhere, even if the part is in great working condition. To get from point A to point B, we need to bring all the pieces together to make an integrated, functional unit.

If nothing else, there is an overwhelming need to recognize complementarity. Thus, we need to move away from the idea of operating in an intradisciplinary manner and push for a sense of transdisciplinarity within the field of proteomics. As we have shown here, all available methods have strengths and weaknesses. However, if we are to work together and take the best qualities of these methods and integrate them, we could well revolutionize the field by fully driving the necessary analyses beyond the current general confines of proteogenomics. Until this is done, we will be unable to advance our methods or thus deep and routine proteomic analyses to the extent that is both necessary and sufficient for unbiased identification of genuine highly selective biomarkers and drug targets. That said, there are indeed critical ongoing efforts to improve both sample and data analyses, and the best of these must be integrated into a continuously developing unified approach to proteoform and thus proteome analysis [29,81,88,257,258,259,260,261,262,263,264,265,266,267,268,269,270,271,272,273,274]. We are hopeful and confident of a more collaborative and unbiased future for the discipline of proteomics.

***For a successful technology, reality must take precedence over public relations, for Nature cannot be fooled*.**

***Richard P. Feynman***
